# Phytochemistry and Biological Activities of Agrostemma Genus—A Review

**DOI:** 10.3390/plants13121673

**Published:** 2024-06-17

**Authors:** Aleksander Smakosz, Adam Matkowski, Izabela Nawrot-Hadzik

**Affiliations:** Department of Pharmaceutical Biology and Biotechnology, Faculty of Pharmacy, Wroclaw Medical University, 50-556 Wroclaw, Poland; aleksander.smakosz@student.umw.edu.pl (A.S.); izabela.nawrot-hadzik@umw.edu.pl (I.N.-H.)

**Keywords:** corncockle, triterpene saponins, ribosome-inactivating proteins

## Abstract

The family Caryophyllaceae comprises more than 2600 species spread widely across all the continents. Their economic importance is mainly as ornamentals (carnation) and as weeds in agriculture. Some species have been used traditionally (and some are still) in herbal medicine or as emulsifiers in food processing. These applications are based on the high content of triterpenoid saponins. Typical for this family are also ribosome-inactivating proteins (RIPs), which are potentially highly toxic. *Agrostemma githago* L. (common corncockle) was historically considered a serious toxicological hazard owing to cereal grain contamination by its seeds. Notwithstanding, it was also recommended as a drug by various herbalists. In this review, the literature was searched in the PubMed, Google Scholar, and Scopus databases for papers focused on the chemical composition and bioactivity of the two accepted species of the *Agrostemma* genus. This systematic review adhered to the Preferred Reporting Items for Systematic Reviews and MetaAnalysis (PRISMA) guidelines. Current research reports the cytotoxicity against neoplastic cells; the protection against oxidative stress; the suppression of *Leishmania major* culture growth; the inhibition of protein synthesis; and the antiviral, anti-angiogenic, and antihypercholesterolemic activities of common corncockle. The future prospects of using *A. githago* saponins as adjuvants in drug formulations and enhancing the cytotoxicity of RIPs are also discussed.

## 1. Introduction

There are an estimated 374,000 or more plant species worldwide (2016) [1]. Among these, at least 28,187 species are used as a source of raw materials for medicinal resources: 16% of these are standardized and centrally regulated. What is also important, is that 80% of all plant-derived food comes from only 17 plant families [2].

Still, in many parts of the world, medicine is based on traditional systems, in which plants are the main source of raw materials for medicine. This is especially the case in rural areas of Africa, Asia, and Central and South America, where there is easy access to traditional herbal practitioners. In developed countries, ethnopharmacological data are used in two ways. The first model involves the complete integration of official medicine and traditional medicinal systems. This modus operandi is implied in the “WHO Strategy towards Traditional Medicine 2014–2023”. This document also states the need for regulation, clinical research, rationalization, and dissemination of traditional treatments [3].

In the second model, traditional medicine and ethnobotanical work inspire preclinical and clinical research. As a result of the field work, previously unknown plant species and compounds with medicinal potential could be found [4,5]. During the period from 1981 to 2019, 39% of newly registered drugs were originally derived from natural sources. Among small anticancer drugs, 33% were obtained from natural (botanical) sources [6].

A hitherto underestimated plant, rich in constituents that could be used in the development of novel pharmaceutical formulations, is *Agrostemma githago* L. (common corncockle). In recent years, this plant species has become an object of interest in phytochemical and pharmacological research. According to scientific data, it contains two classes of major biologically active compounds, ribosome-inactivating proteins (RIPs) and triterpene saponins.

To date, triterpene saponins have been detected mostly in Dicotyledons. They are present in many medicinal and industrial plants, e.g., *Ipomea batatas* (sweet potato), *Gypsophila* spp. (baby’s breath), *Argentina anserina* (silverweed), *Crocus sativus* (saffron), *Chenopodium quinoa* (quinoa), *Dianthus* spp. (pink/carnation), etc. [7,8,9]. Due to their surfactant properties, saponin-rich plants and pure saponins are still used as foaming agents, solubilizers, cleaning agents, emulsifiers, and wetting agents [10]. In this regard, there are attempts to utilize them in commercial products. In addition to this, saponins have a broad spectrum of activity and thus potential pharmaceutical and cosmetic applications. They are permeability enhancers and anticholesterolemic, antioxidant, antimicrobial, anti-inflammatory, cytotoxic, and hemolytic agents [10]. Saponins are also used as immunological adjuvants—they enhance the immune response to an antigen [11]. The second most important *Agrostemma* constituents not belonging to small-molecular-weight specialized metabolites are ribosome-inactivating proteins (RIPs). This group of compounds also has a broad spectrum of action, e.g., antiviral, antifungal, and insecticidal activities; cytotoxic properties; immunological effects; and abortifacient activities. In part, these properties are explained by the nucleic-depurinating activity of RIPs; they act as adenine polynucleotide glycosylase [12,13]. Some of above effects have been observed in the case of corncockle. Importantly, saponins from corncockle are not as toxic as saponins from other plants, and thus they could be used in novel pharmaceutical formulations [14].

Nevertheless, deeper knowledge of these controversial plants is required. Thus far, there has been no review article on the *Agrostemma* genus. Therefore, we decided to conduct a systematic review on the phytochemistry and biological activity of the *Agrostemma* genus, the results of which are presented in this article. Contemporary research indicates that corncockle can be useful in advanced anticancer therapies as well as novel pharmaceutical preparations. In order to be able to develop these, it is necessary to summarize the state of the art and identify knowledge gaps. We hope that this review will help to plan future studies and rationally utilize this easy-to-grow and promising plant.

## 2. Botanical Profile and Taxonomy

The genus *Agrostemma* (*Caryophyllaceae*) consists of two currently accepted species: *Agrostemma githago* L. and *Agrostemma brachylobum* (Fenzl) K.Hammer (https://powo.science.kew.org, accessed on 4 June 2024).

**Figure 1:**
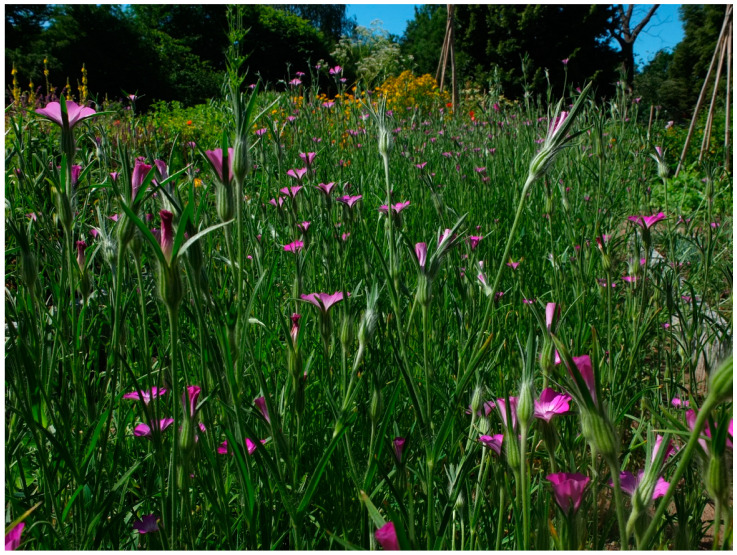
Experimental culture of *A. githago* (Wroclaw Medical University, Poland).

*A. githago* is an annual herb with a 50–150 cm erect stem (Figure 1). It bears 1–10 flowers. They are 2–5 cm in diameter. The leaves are opposite (5–13 cm), acute linear lanceolate, with white hairs. There are three varieties of this species: typical *githago* and large-seed *macrospermum* (Levina) Hammer, both found in Europe’s cereal fields, and small-seeded *linicola* (Terech.) K. Hammer, which has been observed in flax fields of Eastern Europe [15]. In mountainous areas between the Pindos range and the coastal mountains of eastern Central Greece, the endemic *Agrostemma githago* subsp. *thessalum* (Bornm.) Greuter grows (currently, this taxon is classified as an *A. brachylobum* synonym) [16].

**Figure 2:**
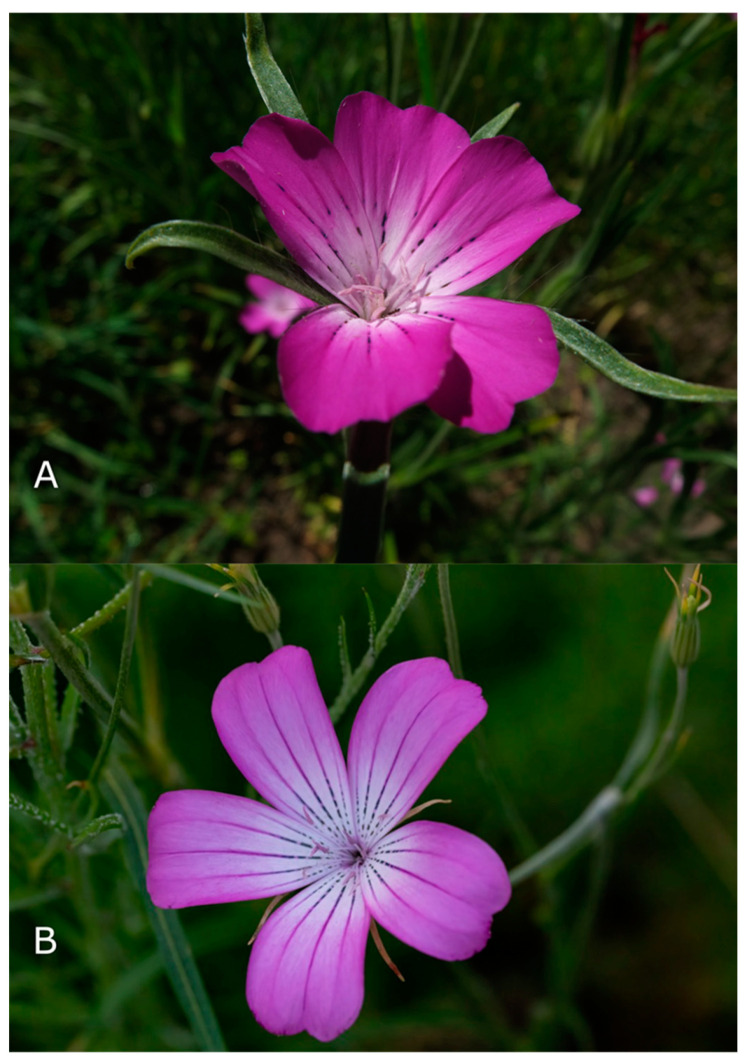
Flower morphology of the two related species: (**A**) *A. githago*, photo by Aleksander Smakosz; (**B**) *A. brachylobum* (attribution *Agrostemma brachyloba* (Fenzl) K. Hammer observed in the Netherlands (Kingdom of the) by Daan Sitters (licensed under http://creativecommons.org/licenses/by-nc-nd/4.0/, accessed on 6 June 2024)). The *A. githago* flower is distinguished by the calyx (green) teeth much longer than the petals, and in *A. brachylobum* they do not reach out of the corolla diameter.

The second species, *A. brachylobum* (=*Agrostemma gracile* Boiss.), is found mainly in Turkey and Greece. It is smaller and less hairy and has a narrower calyx tube and petals longer than the calyx teeth (Figure 2) [15]. Despite the declining occurrence of *A. githago* and a limited geographic distribution of *A. brachylobum*, no conservation status has been classified neither by IUCN nor EUNIS.

The name *Argostemma* was also given to a genus from the *Rubiaceae* family by Wallich (1824). These similar names are sometimes misspelled and should be considered during a literature search to avoid confusion [17].

## 3. Historical Aspects of *Agrostemma githago*

Common corncockle was named *Agrostemma githago* by Carl von Linné in his first edition of Species Plantarum (1753). He enlisted four species: *A. githago* L., *A. caeli-rosa* L. (≡ *Silene coeli-rosa* (L.) Godr.), A. *coronaria* L. (≡ *Silene coronaria* (Desr.) Clairv. ex Rchb.), and *A. flos-jovis* L. (≡ *Silene flos-jovis* (L.) Greuter & Burdet.) [18]. Narrow corncockle (*Agrostemma brachylobum* (Fenzl) K.Hammer) was described for the first time by Edmund Boissier in Diagnoses Plantarum Orientarum Novarum [lat. Diagnoses of new oriental plants]; he collected this species in mountains of Anatolia and Lydia (Turkey) and named *Agrostemma gracilis* Boiss [19]. The name of this genus is derived from Greek: agros (αργοσ) “field” and stemma (στέμμα) “garland, crown”. In older sources, this plant often appeared under other names: “*Caryophyllus arvensis*”, “Anthemon”, “Lichnis/Lychnis”, “*Melanthium agreste*”, “*Nigella arvensis cornuta/silvestris*”, “Nigellastrum”, and “Pseudomelanthium” [20].

The interactions between this taxon and humans could be as ancient as human civilization itself. Some researchers are of the opinion that “cockle” of the Bible is *A. githago*. The Hebrew word employed in this book is “caoshah” or “coash”; the Greek equivalent is “batos”, which corresponds to any “noisesome weed”. In the Book of Iob, the cockle grows instead of barley on the field. *A. githago* was a strong-growing and troublesome plant that grew in the Middle East, which seems that it fits to this Bible context [21]. However, the Poaceae member *Lolium temulentum* matches better with the other biblical context of the cockle—the Parable of the Tares (Matthew 13:30).

We do not know what this plant was called in antiquity and whether it was used medicinally. Some studies say that “Pseudomelanthium” was known by Hippocrates, Theophrastus, Galen, and Pliny the Elder [20,22]. There is no evidence of using corncockle in medieval medicine. The spread of *A. githago* took place at the turn of the 17th century [20]. Therefore, some physicians, like Daniel Sennert (1572–1637), tried to implement this crude drug into official medicine. He used *A. githago* to treat ulcers, fistulas, and hemorrhages. Because of the “miraculous healing” of citizens of Denmark, he was highly regarded as a “magician”. In the following years, *A. githago* gained a great reputation among physicians. Adam Lonicer (1582) proposed a recipe for an antihelmintic plaster containing wormwood (*Artemisia absinthium* L.), honey, and flour prepared by grinding corncockle seeds. He also mentioned that *A. githago* could be used for toothache treatment (acetic extract), for stings from venomous animals, for rashes (ointment), and for limb pain (water herb extract). Other contemporary authors mentioned that seeds of corncockle decocted with wine are efficient against hernias; mixed with “sulphur wine” and vinegar, it healed rashes and coughs [20]. In Traditional Chinese Medicine (TCM), the roots of *A. githago* were known as *Mai Xian Weng* [23]. More recent archaeobotanical studies (2008) evidenced that *A. githago* as well as *Claviceps purpurea* and *Onopordum acanthium* were used (or were present on fields) in the areas of present Austria, Germany, Switzerland, and Poland as long ago as the fourth millennium B.C. [24]. Corncockle seeds contaminate grain; corncockle poisons bread and alters the taste. Some researchers suggest that this altered taste was preferred by Viking communities [25].

The first researcher who chemically analyzed *A. githago* was E. Rülig (1845). During his study, he only measured the content of salts and inorganic acids [26]. One year later, Heinrich Schulze—a pharmacist from Cottbus—published his findings about the isolation of “agrostemmin” (probably a mixture of saponins and proteins). In this case, the pulverized seeds of *A. githago* were extracted with 40% alcohol acidified with acetic acid, filtered, then heated with magnesium oxide. In the last step, the precipitate was filtered off, dried, and extracted with alcohol, from which the “agrostemmin” was crystallized after evaporation and purified by repeated recrystallisation [27]. The above findings inspired E. A. Scharling to publish his work (carried out 17 years earlier) about “githagin” [28]. He developed three methods of isolation. In one of them, he defatted ground seeds, performed an extraction with hot alcohol, filtered the extract, and evaporated the solvent. In the next steps, he added lead(II) oxide and acetic acid [28].

In the following years, this plant continued to be studied, for example by Nicolai Kruskal from Kaunas (Lithuania). In 1891, he published a study about the presence and extraction methods of saponins in various crude drugs (including *A. githago*). In the same volume, he published a monographic article solely about corncockle (*Über Agrostemma githago* L. = *About Agrostemma githago* L.) [20]. Other phytochemical studies were carried out in 1904 by Karl Sänger. He isolated sapotoxin and sapogenin from an ethanolic extract of *A. githago* [29].

Currently, the two main groups of bioactive ingredients from *Agrostemma* spp. are best known and the most intensively studied. They are ribosome-inactivating proteins (RIPs) and triterpenoid saponins. Although the action and isolation of RIPs have been known since the 19th century (pure ricin was isolated for the first time from *Ricinus communis* L. in 1888 by Hermann Stillmark), the classification of these proteins took place in the 1980s [12,30,31].

*Agrostemma githago* was never a pharmacopeial plant, although there are available homeopathic preparations based on corncockle. One Indian manufacturer states that *A. githago* should be used for vertigo, headache, and impaired locomotion [32]. Due to the lack of detailed historical data, any toxicological and pharmacological assessment of *A. githago* crude drugs and extracts is very complicated (also, they are not listed in the Generally Recognized as Safe (GRAS) database). *A. githago* has been extensively studied in terms of its phytochemicals and bioactivity for several years. For this reason, a need came up to provide a review article that would help researchers to understand the state of the art and figure out possible future research directions and potential applications.

## 4. Methods of Systematic Review

### 4.1. Search Strategy

This systematic review adhered to the Preferred Reporting Items for Systematic Reviews and MetaAnalysis (PRISMA) guidelines [33]. An electronic database search was conducted using PubMed, Google Scholar, and Scopus (as of 1 May 2024).

The search terms included all combinations of the following key words:
Database: PubMed((Agrostemma githago) OR (Agrostemma hirsuta) OR (Agrostemma macrospermum) OR (Agrostemma niceaensis) OR (Githago segetalis) OR (Githago segetum) OR (Agrostemma brachylobum) OR (Lychnis githago) OR (corncockle) OR (common corn-cockle)) AND ((phytochemistry) OR (activity) OR (extract) OR (compound))Database: Google Scholar(“Agrostemma githago” OR “Agrostemma hirsuta” OR “Agrostemma macrospermum” OR “Agrostemma niceaensis” OR “Githago segetalis” OR “Githago segetum” OR “Agrostemma brachylobum” OR “Lychnis githago” OR “corncockle” OR “common corn-cockle”) AND (“phytochemistry” OR “activity” OR “extract” OR “compound”)Database: ScopusALL(Agrostemma githago OR “Agrostemma hirsuta” OR “agrostemma macrosperma” OR “agrostemma cearensis” OR “Githago segetalis” OR “Githago segetum” OR “agrostemma brachysporum” OR “Lychnis githago” OR “corncockle” OR “common corn-cockle” AND “phytochemistry” OR “activity” OR “extract” OR “compound”)

All titles with abstracts were imported into a citation manager program, “Mendeley” (Elsevier-Mendeley Ltd., London, UK), and all duplicates were removed. The bibliographies of imported articles were also screened for other relevant studies. Two investigators (A.S. and I.N.-H.) independently reviewed the titles and abstracts of the imported references to determine whether they met the inclusion and exclusion criteria. Disagreements were resolved via consensus and by the third investigator (A.M.).

### 4.2. Inclusion Criteria

The inclusion criteria were as follows: (a) all relevant studies reporting the biological activity of an extract/fraction or a composition of compounds as well as single compounds from *A. githago;* (b) all relevant studies reporting the biological activity of an extract/fraction or a composition of compounds as well as single compounds from *A. brachylobum.* Only studies published in the English language were taken into consideration (non-English papers with factually relevant information or English abstracts were included in this review). All included articles were critically read and analyzed. If there were any uncertainties regarding the quality of a study not filtered out during the preliminary assessment, it is described in this manuscript.

### 4.3. Exclusion Criteria

We excluded the following types of experimental papers: research treating *A. githago* as a weed or on chemicals that have influence on plant growth, cultivation papers, and articles about the biosynthesis of *Agrostemma* constituents.

### 4.4. Data Organization

The authors, year of publication, type of study, type of compound/extract, compound concentration, types of cells and tissues, methods, and principal findings of each study are noted in tables. The studies were divided into two parts: (1) phytochemistry; (2) biological activity. Both parts were divided into subsections.

## 5. Results and Discussion

**Figure 3:**
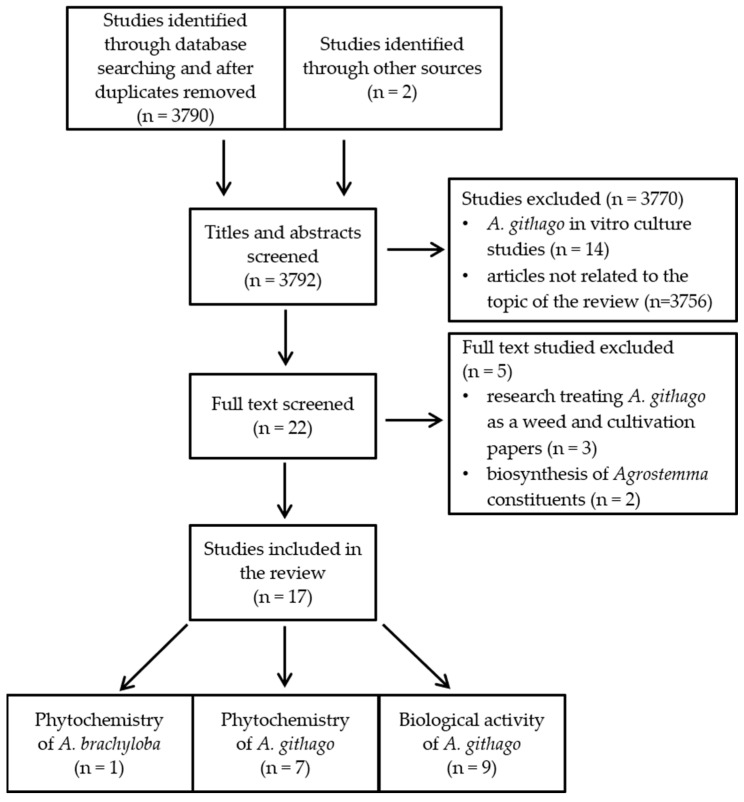
Flowchart of the article search strategy, exclusion criteria, study selection, and data management process.

After duplicate removal, 3792 articles were further screened by title and abstract. Finally, seventeen studies met the inclusion criteria (Figure 3). The quality score of the included bioactivity studies are shown in Table 1.

### 5.1. Phytochemistry of A. githago and A. brachyloba

Until now, five saponins named agrostemmosides have been isolated from *A. githago* (from agrostemmoside A to agrostemmoside E). In addition to agrostemmosides, four other saponins have also been isolated (not included in agrostemmoside series). The aglycons of the above compounds are derivatives of gypsogenin (3-beta-hydroxy-23-oxoolean-12-en-28-oic acid) and quillaic acid (3,16-dihydroxy-23-oxoolean-12-en-28-oic acid). Niapour et al. (2023) isolated five new triterpenoid saponins from seeds (calculated masses: 745.13; 869.67; 907.81; 907; 928) but with unknown structures. In the same study, the researchers measured the total phenolic and flavonoid contents of *A. githago* seeds (17.75 ± 1.21 mg/100 g and 4.02 ± 0.12 mg/100 g, respectively) [43]. Similar saponins occur in *Gypsophila* spp. [44].

**Figure 4:**
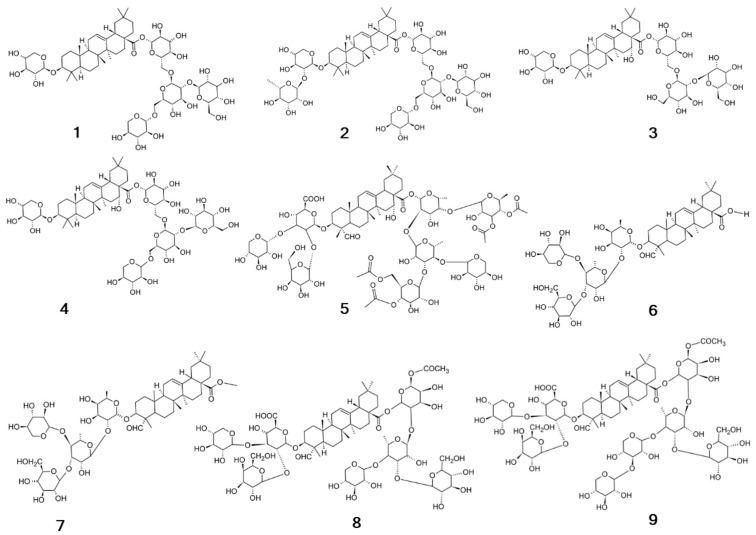
Structure of isolated saponins. The structures were drawn using ChemDraw 22.2 (Perkin-Elmer, Shelton, CT, USA) [47]. The numbers correspond to the following compounds: 1. Agrostemmoside A: 3-O-β-D-xylopyranosyloleanolic acid 28-O-β-D-glucopyranosyl-(1 → 2)-[β-D-xylopyranosyl-(1 → 6)]-β-D-glucopyranosyl-(1 → 6)-β-D-glucopyranosyl ester. 2. Agrostemmoside B: 3-O-α-L-rhamnopyranosyl-(1 → 2)-β-D-xylopyranosyloleanolic acid 28-O-β-D-glucopyranosyl-(1 → 2)-[β-D-xylopyranosyl-(1 → 6)]-β-D-glucopyranosyl-(1 → 6)-β-D-glucopyranosyl ester. 3. Agrostemmoside C: 3-O-β-D-xylopyranosylechinocystic acid 28-O-β-D-glucopyranosyl-(1 → 2)-β-D-glucopyranosyl-(1 → 6)-β-D-glucopyranosyl ester. 4. Agrostemmoside D: 3-O-β-D-xylopyranosylechinocystic acid 28-O-β-D-glucopyranosyl-(1 → 2)-[β-D-xylopyranosyl-(1 → 6)]-β-D-glucopyranosyl-(1 → 6)-β-D-glucopyranosyl ester. 5. Agrostemmoside E: 3-{O-ß-D-Galactopyranosyl-(1→2)]-[ß-D-xylopyranosyl-(1→3)]-ß-D-glucuronopyranosyl} quillaic acid 28-O-{[ß-D-4,6-di-(O-acetyl)-glucopyranosyl-(1→3)]-[ß-D-xylopyranosyl-(1→4)]-α-L-rhamnopyranosyl-(1→2)}-[3,4-di-(O-acetyl)-ß-D-quinovopyranosyl-(1→4)]-ß-D-fucopyranoside ester. 6. 3-O-[-ß-D-glucopyranosyl-(1→3)-ß-D-xylopyranosyl-(1→4)-α-L-rhamnopyranosyl-(1→2)-ß-D-fucoopyranosyl]-3ß-hydroxy-23-oxo-Δ-12-oleanen-28-carboxylic acid. 7. 3-O-[-ß-D-glucopyranosyl-(1→3)-ß-D-xylopyranosyl-(1→4)-α-L-rhamnopyranosyl-(1→2)-ß-D-fucoopyranosyl]-3ß-hydroxy-23-oxo-Δ-12-oleanen-28-carbonyloxy methyl. 8. Saponin isolated by Siepmann et al. [47]: 3-O-β-D-xylopyranosyl-(1→3)-[β-D-galactopyranosyl-(1→2)]-β-D-glucuronopyranosylgypsogenin-28-O-β-D-xylopyranosyl-(1→4)-[βD-glucopyranosyl-(1→3)]-α-L-rhamnopyranosyl-(1→2)-β-D-4-O-acetylfucopyranoside. 9. Saponin isolated by Siepmann et al. [47]: 3-O-β-D-xylopyranosyl-(1→3)-[β-D-galactopyranosyl-(1→2)]-β-D-glucuronopyranosylgypsogenin-28-O-β-D-xylopyranosyl-(1→3)-β-D-xylopyranosyl-(1→4)]-[β-D-glucopyranosyl-(1→3)]-α-L-rhamopyranosyl-(1→2)-β-D-4-O-acetylfucopyranoside.

An overview of the phytochemistry of *A. githago* and *A. brachylobum* is presented in Table 2. The structures of all the saponins isolated so far are presented below in Figure 4.

**Table 2:** Phytochemistry of *A. githago* and *A. brachylobum*.

Group of Compounds/ Compound	Species	Part of Plant	Isolation/Identification Method	No. *	Ref.
Triterpenoid saponins	*A. githago*	seeds	A mixture of ground seeds and water was homogenized with an ultrasonic homogenizer. The centrifuged extract was lyophilized and analyzed with HPLC.	5	[43]
Triterpenoid saponin agrostemmoside E	*A. githago*	seeds	A dry methanolic extract was fractionated by size-exclusion chromatography and HPLC.	5	[42]
Triterpenoid saponins (agrostemmosides (A–D))	*A. brachylobum*	whole plant/herb	The methanol extract of the whole plant was fractionated over Sephadex LH-20. The saponin fraction was partitioned by column chromatography.	1–4	[45,46]
Githagenins/gypsogenins	*A. githago*	seeds	An extraction was performed with 50% methanol, column chromatography, and elution with methanol and water. The methanol fraction was chromatographed on silica gel with CHCl_3_:CH_3_OH:H_2_O (6:3:1) as the eluent.	8–9	[47]
Githagosides, githagenins/gypsogenins	*A. githago*	seeds	An extraction was performed with 50% methanol, column chromatography, and acid hydrolysis.	6, 7	[48]
Ribosome-inactivating proteins (RIPs) —agrostin	*A. githago*	seeds	An extraction was performed with phosphate-buffered saline and a protease inhibitor. In the next step, separation was carried out by affinity chromatography using an anti-agrostin antibody.	—	[41]
Ribosome-inactivating proteins (RIPs)	*A. githago*	seeds	An extraction was performed with NaCl, a sodium phosphate buffer, and column chromatography (CM-cellulose column; eluent: NaCl in phosphate buffer).	—	[14]

* the numbers in the column correspond to the structure labels in Figure 4.

The first researchers who described the structure of any *A. githago* saponin (structures 6 and 7 in Figure 4) were Tschesche and Schulze (1974) [48]. Their article was written in German (with an English abstract). The aim of their research was to isolate and determine the main saponin from *A. githago* seeds. After extraction with methanol, they defatted the extract with petroleum ether. The extract was chromatographed on silica gel. The structure was determined by an NMR (nuclear magnetic resonance) analysis after hydrolysis and the methylation of sugars.

In the previous research papers, the most commonly used solvents to extract bioactive molecules from *A. githago* were methanol and water (also mixture of both). This limited approach explains why only RIPs and saponins are noted in the available scientific literature. Very often, the studied extracts lack defined phytochemistry [36,37,38,40].

The first English-language article dedicated to agrostemma saponins was published by Siepmann et al. (1998) [47]. Former articles dedicated to this group of secondary metabolites were written in German [48]. In Siepmann’s article [47], researchers isolated two new saponins (derivatives of acetylfucopyranoside). They obtained these molecules from *A. githago* seeds via extraction with 50% methanol after defatting the plant material with petroleum ether. The concentrated extracts were chromatographed on Diaion HP-20. The obtained fractions were purified using column chromatography (silica gel 60, 0.063—0.2 μm).

Niapour et al. (2022) used water as an extraction solvent. After using an ultrasonic homogenizer, they centrifuged the insoluble seed material and collected the supernatant. In the next step, they freeze-dried the extract to obtain a dry extract. The chromatographic profile and LC-MS data enabled the detection of five saponins, and one of these was agrostemmoside E (calculated mass: 1856/2 = 928). However, the identification was based only on the MS profile [43].

Koz and his research group published various research on *Agrostemma* spp. In one conference abstract [45], they analyzed a methanol extract (later treated with hexane) of the *A. brachylobum* herb. After fractionation over Sephadex LH-20, they purified saponin fractions and obtained oleane-type saponins. In the next step, they analyzed the isolated saponins with NMR and described the structures of four triterpene saponins [46].

A similar method was used by Clochard et al. (2020). They extracted previously defatted ground seeds with 90% methanol. After lyophilization, selected fractions obtained after separation of the extract using medium-pressure chromatography (Sephadex LH-20) were subjected to semi-preparative HPLC. The structures of obtained isolates were determined with 1D- and 2D-NMR spectroscopy. As a result, they characterized agrostemmoside E for the first time [42].

Initially, the toxicity of *A. githago* was only attributed to triterpene saponins, but the understanding of this problem has changed thanks to the research of Stirpe et al. (1983). Three ribosome-inactivating proteins from *A. githago* were isolated via the extraction of seeds in phosphate buffer. To isolate the RIPs, they performed single-step chromatography on CM-cellulose [14]. 

A different method of RIP isolation was applied by Weise et al. (2020). They isolated agrostin (one of the RIPs) from a seed aqueous extract using affinity chromatography (the anti-agrostin antibodies were used). The structure of the RIPs was studied using MALDI-TOF–MS [41].

### 5.2. Biological Activity of A. githago and A. brachyloba

**Figure 5:**
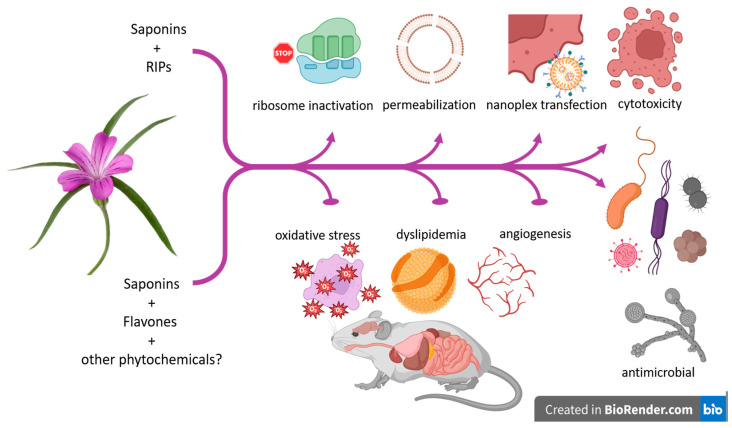
Infographics showing properties of *A. githago*.

An overview of the biological activity of *A. githago* is presented in Table 3 and illustrated in Figure 5.

#### 5.2.1. Cytotoxicity

By now, six studies have been reported on the cytotoxicity of *A. githago* extracts, fractions, or isolated agrostin (RIP type 1) against tumor cells: a bladder carcinoma cell line (ECV-304) [35,41], murine neuroblastoma cell line Neuro-2A (ATCC^®^CCL-131™) [42], human hepatocellular carcinoma cell line HuH-7 (JCRB0403), a gastric cancer cell line (AGS) [38,39], a B-cell lymphoma 2 (Bcl-2) cell line, a human urinary bladder carcinoma cell line, and a human leukemic HL-60 cell line [34].

To date, no report has been found in the literature on the potential cytotoxicity of compounds or extracts from *A. githago* towards a normal cell line.

##### Ribosome-Inactivating Proteins

Ribosome-inactivating proteins have been known since 19th century [30]. However, it was not until the second half of the 20th century that their structure and pharmacological properties began to be studied in detail [31,49]. Type 2 RIPs (e.g., ricin and abrin) are composed of two polypeptide chains: an enzymatic A chain and a lectinic B chain [50,51]. Type 1 RIPs are less toxic because they do not bind to cells easily (they lack the lectin subunit) [34].

These toxins destroy about 2000 ribosomes per minute after entering the cell, and thus they block protein synthesis [52]. Ribosome-inactivating proteins are transported into the ribosome via the following pathway [52,53].

The RIPs bind to glycoproteins and glycopeptides with terminal galactose (membrane receptor site); simultaneously, the carbohydrate side chains are recognized by cell membrane receptors:

The caveolae transport the RIPs during endocytosis.
Toxins move from the endosomes to the Golgi apparatus.
In the Golgi apparatus, RIPs are transported in COPI-coated vesicles.
The chain of type 1 RIPs damages the ribosomes through its enzymatic activity: N-glycosidase at a specific site (adenine).
The larger subunit of ribosomes become unable to bind the elongation factors.
The production of proteins is arrested.

Numerous type 1 RIPs isolated from plant material have been tested with some success on cancerous cell lines. Trichosanthin (isolated from *Trichosanthes kirilowii* Maxim.) at a concentration of 12.5 μg/mL is cytotoxic to leukemia and lymphoma cell lines. The described compound inhibited the proliferation of cancer cells and induced apoptosis [54]. The MAP30 RIP (isolated from *Momordica charantia* L.) inhibited cell viability in HepG2 (human liver cancer cell line) in a time- and dose-dependent manner. The apoptosis of these cells was induced by the activation of the caspase-8 and caspase-9 signaling pathways [55]. Another example is cucurmosin (isolated from *Cucurbita moschata* Duchesne ex Poir.), which also induced apoptosis in the HepG2 cell line via activation of caspase-3 and G0/G1 interface arrest [56]. Interestingly, the cytotoxicity of type 1 RIPs is enhanced by saponins [57].

One of the most important limitations in drug formation technology is low API (Active Pharmaceutical Ingredient) lipophilicity; therefore, drugs have difficulty in crossing cell membranes. One of the possible technological solutions is to insert the active substance or an extract inside the lipid vesicles, such as liposomes. The advantage of using these spherical vesicles is low cost, similarity (the morphology) to cellular membranes, and the ability to incorporate both lipophilic and hydrophilic substances [58]. 

Bohlooli and Fathi [38] encapsulated a lyophilized aqueous extract of *A. githago* seeds and tested it on gastric cancer cells. The IC_50_ values showed that the extract in the liposomes is statistically significantly more active than the pure extract (4.43 ± 1.49 μg/mL vs. 13.02 ± 0.95 μg/mL). The liposomal form is more efficient for delivering the active ingredients (RIPs and triterpene saponins) to cells. The probable mechanism responsible for this effect is boosting the saponin ability to enhance the agrostin entrance into the cells. In this publication, we found a potential flaw in the methodology. The studied extracts were not tested for bioactive compounds: only the content of the extract in liposomes was determined by measuring the UV-Vis absorption of flavonoids. The lack of a phytochemical analysis hinders the potential implementation of the above formulation in clinical practice. 

In another study carried out on HeLa cells, RIPs isolated from *A. githago* inhibited poly(U)-directed polyphenylalanine synthesis by purified reticulocyte ribosomes. This phenomenon occurred both when the proteins (RIPs) were present in the reaction mixture and when the ribosomes were preincubated with the proteins and washed before the assay. The percentage of inhibition reached for the three tested proteins, in comparison to the control sample, was 19%, 28%, and 25%, respectively. The tested RIPs showed no RNAase or proteinase activity. The ID_50_ was >100 μg/mL for all the tested ribosome-inactivating proteins [14].

Chiu et al.’s study showed that the probable mechanism responsible for apoptosis induction by *A. githago* RIPs is the down-regulation of the intracellular bcl-2 (B-cell lymphoma 2) protein level by agrostin [34]. This study showed that in addition to the regulation of protooncogenes, the agrostin inhibits the ^3^[H]-thymidine incorporation, which results in retardation of proliferation, so the RIPs isolated from the *A. githago* (agrostin) could be used as a potential conjugate of immunotoxins [34].

##### Saponins

Clochard et al. (2020) isolated agrostemmoside E from *A. githago* to use it as a transfection modulator for synthesized nanoplexes [42]. Nanoplexes are nanoparticle complexes with an oppositely charged polyelectrolyte. The advantage of this type of particle is the portability of a cationic or anionic molecule. They bind to the oppositely charged polyelectrolytes, thereby increasing their solubility in the aqueous phase and their bioavailability [59]. Agrostemmoside E was demonstrated to have low toxicity and to be an effective transfection enhancer. No harmful effects in the cells were observed up to the concentration of 24 μg/mL [42]

Weise et al. (2020) investigated the cytotoxic effects of agrostin combined with saponin extracted from *Saponaria officinalis* L. on bladder carcinoma cells (cell line ECV-304). The study showed that saponin enhanced the cytotoxicity of RIP by improving the delivery of the protein to the ribosomes [41]. In another study, the incubation of the human neuroblastoma cell line with agrostemmoside E led to reduced viability but no toxicity. The incubation of both the above cell lines with *G. elegans* saponin (24 μg/mL) and puromycin (4 μg/mL) showed significantly stronger toxicity [42].

Considering the preceding reports, it can be concluded that triterpene saponins have moderate cytotoxic properties against various neoplastic cell lines [42]. Thus far, no studies on the cytotoxicity of pure saponins on normal cells lines have been published. The agrostemmasaponins (glycosides of gypsogenin, quillaic acid, and their derivatives) have cytotoxic properties of a similar order of magnitude to other *Caryophyllaceae* taxa. These saponins increase RIP cytotoxicity against neoplastic cell lines [41]. More research on the cytotoxic activity of pure saponins against normal and neoplastic cells is required.

The above findings should come as no surprise, because most species of the *Caryophyllaceae* family are a rich source of triterpene saponins. The cytotoxic effects of saponins isolated from these plants have been repeatedly described in other papers [60]. Some of them are presented below.

From the nine compounds extracted from *Dianthus versicolor* Fisch. ex Link., the most potent was dianvericoside C. Its IC_50_ (tested on various cell lines) varied between 2.9 and >10 μg/mL. The above compound inhibited to the greatest extent HFL-I (human fetal lung fibroblast) and BGC-803 (human gastric cancer cell lines). The IC_50_ was 3.2 and 3.1 μg/mL, respectively [61]. Arslan et al. (2012) isolated a quillaic acid bidesmoside from the roots of *Gypsophila pilulifera* Boiss. & Heldr and showed its weak in vitro cytotoxicity (at the concentration of 1 mg/mL) against A549 (lung carcinoma cell line). The IC_50_ was calculated as 16.5 μM (equivalent of 28.05 μg/mL) [62]. The lyophilized crude extract (10% aqueous methanol) from *Gypsophila trichotoma* Wender roots was tested against NR8383 (rat alveolar macrophage-like cell line), U937 (human leukemic cell line), and BV-173 (chronic myelogenous leukemia cell line). The cytotoxicity was higher to human leukemic cell lines U937 (IC_50_ = 10.97 μg/mL) and BV-173 l (IC_50_ = 40.9 μg/mL) than to NR8383 (IC_50_ > 100 µg/mL) [63]. Manase et al. (2013) isolated five saponins from *Polycarpaea corymbosa* Lamk. var. *eriantha* Hochst (Oldman’s Cap). They tested them against SW480 (collateral human cancer cell line), DU145 (prostate human cancer cell line), and EMT6 (mammary mouse cancer cell line). “Saponin 1” was the only active constituent with cytotoxicity against the above cell lines, with IC_50_ values ranging from 4.61 to 22.61 μM (SW480: 4.61 ± 2.24 μM; DU145: 17.82 μM; EMT6: 22.61 μM) [64]. Dianversicoside C, isolated from *Dianthus versicolor* Fisch. ex Link, had cytotoxic effects on normal cells, e.g., on HFL-I (human fetal lung fibroblasts) and EVC-304 (human umbilical vascular endothelial cells 304), with IC_50_ = 3.2 μM and 3.6, respectively [61].

In conclusion, a vast amount of data exist pertaining to the cytotoxicity of *Agrostemma* and other Caryophyllaceae plants. However, there are huge differences between models and experimental procedures, resulting in difficulty in reaching reasonable conclusions as to whether or not these properties would indeed be pharmacologically relevant for anticancer therapies. Instead, these results accumulate steadily, which could lead to a better understanding of cellular mechanisms and possible structure–activity relationships. This is a prerequisite of rationally conducted lead discovery and full exploitation of the hidden potential of Caryophyllaceae.

#### 5.2.2. Protective Effect against Oxidative Stress

Küçükkurt et al. (2011) tried to determine the possible protective effects of *A. githago* extracts against the oxidative damage induced by irradiation in Wistar rats. In this study, aerial parts were extracted with ethyl acetate, and the solvent was evaporated. In the next step, the residue was dissolved with methanol, and saponins were suspended over the methanol and then taken from the solution. Rats received the *Agrostemma* extract via gastric gavage at doses of 100 or 200 mg/kg for 20 days and were then exposed to X-radiation. Blood samples from all rats were collected on day 21, and the oxidant status was determined by malondialdehyde, glutathione, ascorbate, β-carotene, retinol concentrations, and total antioxidant ability (AOA). Rats were treated with *A. githago* extracts (100 or 200 mg/kg/day). The most decreased blood biochemistry was (at both concentrations) of glucose, triglycerides, and malonaldehyde. The levels of β-carotene, retinol, and reduced glutathione increased in a significant manner [37]. 

The description of the extraction procedure used in this study is insufficient, so the compounds contributing to these activities could not be suggested. However, the use of ethyl acetate and redissolving in methanol would rather point to something other than saponins or RIP compounds as the active principle. Many Caryophyllaceae, including *A. githago*, are also rich in flavones (mostly C-glycosides), so the possibility that these compounds cause such an effect warrants further investigations.

#### 5.2.3. Antiviral Activity

RIPs isolated from an *A. githago* extract mixed with the tobacco mosaic virus (Virgaviridae) before infection reduced the number of local lesions on the leaves of *Nicotiana glutinosa* L. The observed inhibitions of disease progression of the three isolated RIPs (at a concentration of 50 μg/mL) were 58, 99, and 97%, respectively [14].

The above study is the only research that has focused on the antiviral properties of *A. githago* RIPs. There are numerous publications that describe constituents from other Caryophyllaceae species that inhibit the replication of virions [13]. 

The saporin (RIP) isolated from *Saponaria officinalis* inhibits HIV-1 reverse transcriptase, HIV-1 protease, and HIV-1 integrase (24.2 ± 11.2%, 15.7 ± 9.0%, and 0.9 ± 1.3%, respectively) [65]. Other groups of HIV-inhibiting proteins were isolated from *Dianthus caryophyllus*. The ID_50_ (concentration that reduces the DNA by 50% compared with the virus control) of its RIP (trichosanthin) has a value ranging from 0.34 to 0.46 nM [66]. Also, it was proven that the tobacco mosaic virus is inactivated by extracts from the above species [13]. Tobacco mosaic virus RNA is depurinated completely when mixed with 100 ng of saporin [67]. Dianthin 30 and dianthin 32 (isolated from *D. caryophyllus*) prevented local lesions in the leaves by more than 50% at concentrations of 0.5 and 1 μg/mL, respectively. Other undescribed compounds isolated from this taxon also inhibit the tobacco mosaic virus, but at a higher concertation of 50 μg/mL [68].

#### 5.2.4. Antiprotozoal Properties

In one study [40], the researchers tested the in vitro anti-leishmaniasis (*Leishmania major* promastigotes) potential of an *A. githago* aqueous extract. The IC_50_ value of the *A. githago* extract was 0.365 mg/mL (*p* < 0.05). The inhibitory effect of the extract was stronger than that of the positive control (meglumine antimoniate), but the active substances were not defined, whether it was saponins, RIPs, or both in synergy. RIPs such as dianthins and saporins (from *D. caryophyllus* and *S. officinalis*, respectively) inactivate the ribosomes of Leishmania sp. (IC_50_ ranging from 27 to 110 nM for *D. caryophyllus* and IC_50_ ranging from 26 to 116 nM for *S. officinalis*) [69], but purified RIPs from *A. githago* remain to be investigated for such properties.

#### 5.2.5. Antihypercholesterolemic Activity

The cholesterol-lowering activity of saponins is the aim of the research from the 1950s. Contemporary studies have shown that the metabolites of saponins interact with cholesterol and cholic acid. It is proposed that saponins form insoluble complexes with cholesterol. Further, they form complexes with other food components and alter the cholesterol transporters and may modulate the expression of regulatory genes related to cholesterol metabolism [70].

Avci et al. [36] administered ethanolic and aqueous extracts from the aerial part of *A. githago* to male Swiss albino mice fed with a high-cholesterol diet. Extracts were given orally in 100 mg/kg doses after suspending them in a mixture of distilled water and 0.5% sodium carboxymethyl cellulose via gastric gavage. In this study, the hypercholesterolemic group of the tested animals (fed with fodder containing 1% cholesterol for 30 days) was used as the positive control, and the group maintained on a standard diet and water was used as the negative control. The ethanolic extract of *A. githago* decreased the serum total cholesterol concentration (*p* < 0.05) from 218.4 ± 16.3 mg/dL to 97.2 ± 11.2 mg/dL and the LDL-C concentration (*p* < 0.05) from 143.0 ± 25.3 mg/dL to 42.0 ± 12.0 mg/dL in the mice fed with the high-cholesterol diet. The aqueous extract increased the HDL-C concentration (*p* < 0.001) from 25.8 ± 0.8 mg/dL to 41.5 ± 1.8 mg/dL. The extracts did not have a significant effect on the serum biochemical parameters. The antihypercholesterolemic effect results from the phenomenon of saponin micelle formation with sterols. Cholesterol in this state is not able to be reabsorbed, and thus the excretion is increased [36]. In this case, the absorption of cholesterol into the general circulation will be significantly impaired, and thus the blood level will fall over time. In this study, the extracts also remained uncharacterized, and as such, the attribution of the positive effects in dyslipidemia cannot be solely attributed to saponins. To clarify the contribution of triterpenoids and flavonoids to the final bioactivity, an additional screening using bioguided fractionation would be recommended.

### 5.3. Toxicity of A. githago and A. brachylobum

The toxicity of *Agrostemma* spp. parts depends on two groups of bioactive constituents: RIPs (ribosome-inactivating proteins) and saponins. The toxicity of triterpene saponins is very variable. It depends on polarity, the shape of the compound, and the length of the sugar chain. Monodesmosidic saponins show higher toxicity than their bidesmosidic counterparts. Although they have well-established toxic properties, numerous saponins occur in edible plants (e.g., soya—*Glycine max* (L.) Merr., fenugreek—*Trigonella foenum-graecum* L., and quinoa—*Chenopodium quinoa* Wild.) [9,71].

Hebestreit et al. showed that saponins isolated from a related plant—*Gypsophila* sp. (*Sapopninum album* GS supplied by Merck)—enhanced the cytotoxicity of a type 1 RIP (saporin) from *Saponaria officinalis* 100,000-fold [57]. This demonstrates how important the synergy of RIPs and saponins is in the toxicity of plants from *Caryophyllaceae*.

In another study, researchers tested a saponin- and terpene-rich alcohol–water extract from *Corrigiola telephiifolia* Pourr. (*Caryophyllaceae*). They tried to estimate its toxic profile in rodents. It was shown that a dose of 2000 mg/kg/day increased the concentrations of creatinine (26.4 ± 5.1 to 87.0 ± 15.6 μmol/L), alkaline phosphatase (121.7 ± 10.8 to 202.1 ± 30.0 UI/L), *gamma*-glutamyltransferase (1.4 ± 0.1 to 1.9 ± 0.2 UI/L), and phosphorus (3.2 ± 0.2 to 4.3 ± 0.4 mmol/L) in serum [72].

The proteins isolated from the aqueous extract of common corncockle seeds did not cause any harm when injected into mice at doses up to 1 mg/kg body weight. During the same study, RIPs from *Saponaria officinalis* L. killed mice within 6 days at an LD_50_ of 4 mg/kg [14]. The above data show that *Caryophyllaceae* plants synthesize RIPs with variable toxicity (from very toxic to low toxic/nontoxic). It appears that in the case of corncockle, the use of extracts rich in saponins and low in RIPs can broaden its universal applications (e.g., enhancing the bioavailability). On the other side, RIPs could be potentially used as cytotoxic agents. More research is needed to obtain data about interactions between ribosome-inactivating proteins and saponins and their action on certain cell lines as well as pathogenic microorganisms or parasites. In such studies, not only extracts and fractions should be investigated but also as many as possible isolated compounds and their reconstituted mixtures to fully account for expected synergies.

Transfection enhancement, induced by the impact of agrostemmoside E on cells, was most observable at a concentration of 4 μg/mL (the higher concentrations have no significant differences). On the other hand, the agrostemmoside E-mediated transfection at a high concentration (24 μg/mL) achieved a high transfection efficiency. The tested structure-related triterpene saponins from *Gypsophila elegans* M. Bieb reached no significant transfection increase, due to the toxicity. A human hepatocellular carcinoma cell line incubated with various concentrations (2 to 24 μg/mL) of agrostemmoside E showed no toxic effects [42].

The γ-secretase inhibitor reverses the toxic effect of *A. githago* extracts (reduction in reactive oxygen species and alleviation of the impeded capillary tube-like formation); therefore, it could be used as a regulatory factor of *A. githago* saponins [43,57].

As many other representatives of *Caryophyllaceae*, *Agrostemma githago* exhibits toxic potential that can be considered as a potential limitation to using it as a medicinal plant. This is caused by two classes of compounds: RIPs and triterpenoid saponins. However, due to a lack of reports on other groups of constituents and in vivo toxicological studies, it is difficult to evaluate the true toxicological properties of *A. githago*. Therefore, other interactions than those between RIPs and saponins cannot be excluded. Moreover, despite the huge amount of data on the cytotoxicity of saponins from Caryophyllaceae, a significant ambiguity of these results necessitates deeper insight into the mechanisms of interactions with cell membrane sterols and structure–activity relationships. One of the important dilemmas is, for example, to elucidate the roles of aglycon and sugar moieties in the determination of both permeabilization and other interactions [73]. It is anticipated that future research will help to clarify these issues.

## 6. Conclusions

*Agrostemma githago* has been an important plant both in culture and agronomy. However, it has never been an important medicinal plant, but in the past, it was used for dermal disorders and intestinal worms. Contemporary research indicates that it can be a source of compounds useful in advanced anticancer therapies and a therapeutic agent against various disorders.

Among the reviewed studies, six reported cytotoxicity of *A. githago* against cancer lines [34,35,38,39,41,42]. Three studies were performed on aqueous seed extracts (including one on a nanoliposomal formulation of a seed aqueous extract [37]), two on ribosome-inactivating proteins (agrostin sodium salt and pure agrostin), and one on a seed ethanolic extract and PD-nanoplexes and methanolic extract formulation. Other studies included protective effects against radiation-induced oxidative stress (n = 1 [37]), antiviral effects (n = 1 [14]), the inhibition of *Leishmania major* culture growth (n = 1 [40]), the inhibition of protein synthesis (n = 1 [14]), anti-angiogenic effects (n = 1 [43]), and antihypercholesterolemic activity (n = 1 [36]). One investigation [37] suggested that the encapsulation of an extract may enhance cellular uptake and offer the following advantages (in comparison to a crude extract): longer duration of action, smaller therapeutic dose, and improved bioavailability.

Hence, there is a need for more research, especially combining a reliable phytochemical characterization with a mechanistic approach to bioactivity in vitro and in vivo. A large unexplored area is using in silico methods to accelerate the understanding of the chemical, physicochemical, and molecular basis of the experimentally discovered properties.

The cytotoxic properties of *A. githago* are most likely associated with RIPs or the synergistic action of RIPs and saponins. On the other hand, reports about cytotoxicity on normal (non-transformed) cells of *Agrostemma* saponins are not available, so these compounds should be tested in the future to verify their safety before any clinical applications follow.

Some of the previously reported bioactivities (antiprotozoal, antihypercholesterolemic, antioxidant/radioprotective, antiviral, etc.) could be associated with other phytochemicals, such as flavonoids. The latter are also abundant in the *A. githago* herb [74].

Finally, the centuries-long interest in this plant as a weed or as a mostly anecdotical poisonous and medicinal herb has found its culmination in very promising pharmaceutical and medicinal potential. Therefore, new opportunities have emerged to initiate thorough phytochemical, molecular, and pharmacological investigations aimed at obtaining the full picture necessary for its practical implementation into pharmacy and medicine.

## Figures and Tables

**Table 1:** Comparison of research quality indicators in the studies selected for review. Every indicator (first column) was assigned a point for presence: “1”—present and “0”—absence in the experimental design. The bottom line is a sum of scores that shows the inequal quality of the experimental studies.

	Stirpe et al., 1983 [14]	Chiu et al., 2001 [34]	Hebestreit and Melzig 2003 [35]	Avci et al., 2006 [36]	Küçükkut et al., 2011 [37]	Bohlooli and Fathi 2015 [38]	Bohlooli et al., 2015 [39]	Niapour et al., 2018 [40]	Weise et al., 2020 [41]	Clochard et al., 2020 [42]	Niapour et al., 2022 [43]
Defined cell line/animal	1	1	1	1	1	1	1	1	1	1	1
Defined composition of extract	1	1	1	0	0	0	0	0	1	1	1
Control group	1	1	1	1	1	1	1	1	1	1	1
Statistical analysis	1	1	1	1	1	1	1	1	0	1	1
Points	4	4	4	3	3	3	3	3	3	4	4

**Table 3:** Summary of studied bioactive properties of corncockle.

Properties of *A. githago*	Source (If Available)/ Active Compound/ Extract/Fraction	Results	Ref.
Cytotoxicity against cancer lines	RIP (agrostin) isolated from seeds	The saponin isolated from *S. officinalis* enhanced the cytotoxicity of agrostin against bladder carcinoma cells line (ECV-304) by improving its delivery to the ribosomes.	[41]
Cytotoxicity against cancer lines	Seed ethanolic extract, formulation of methanolic extract and PD-nanoplexes	Formulations with 12 and 24 μg/mL of the *A. githago* seed extract and PD-nanoparticles resulted in a slight decrease in the proliferation of murine neuroblastoma cell line Neuro-2A (ATCC^®^CCL-131™) and the human hepatocellular carcinoma cell line HuH-7 (JCRB0403).	[42]
Cytotoxicity against cancer lines	Nanoliposomal formulation of seed aqueous extract	A water extract from *A. githago* seeds was included into nanoliposomes. The IC_50_ values of an MTT assay of loaded nanoliposomes and the crude extract against a gastric cancer cell line (AGS) were 13.26 ± 1.31 and 4.3 ± 0.64 μg/mL, respectively. Similar IC_50_ values were recorded for the Neutral Red Assay (15.29 ± 0.94 and 5.6 ± 0.59 μg/mL) and FRAME (Fund for the Replacement of Animals in Medical Experiments) assay (10.53 ± 0.61 and 3.41 ± 0.79 μg/mL). The value of the total IC50 was 13.02 ± 0.95 μg/mL for the base extract and 4.43 ± 1.49 μg/mL for the extract-loaded nanoliposomes.	[38]
Cytotoxicity against cancer lines	Aqueous seed extract	The extract was cytotoxic to AGS after 24, 48, and 72 h of incubation. The IC_50_ values were 13.51 ± 0.7, 4.37 ± 1.01, and 2.42 ± 0.8 μg/mL, respectively. The extract induced apoptosis of cells in a dose-dependent manner in all tested concentrations (50–200 μg/mL). The AGS cells treated with the extract had morphological changes representative of apoptosis, revealed by EB/AO staining. The extract-treated cells increased the G1 phase population, while the cell number in the G2/M phase was decreased (analyzed by flow cytometry). The level of the BCL-2 protein in cells treated with the *A. githago* water extract was decreased in a dose-dependent manner. The same correlation was observed with caspase 3 activity. The extract of *A. githago* was able to freeze the cell cycle at the G1 check point.	[39]
Cytotoxicity against cancer lines	Aqueous seed extract, 50% aqueous-methanolic extract, agrostemmasaponin fraction, agrostemmasaponin 1 and 2, helianthussaponin	The combination of agrostemmasaponin 1 with agrostin had the highest cytotoxic properties among all tested constituents and extracts on human urinary bladder carcinoma cells (IC_50_ = 5.7 ng/mL). The cell proliferation was inhibited both by the aqueous and methanolic extracts and agrostemmasaponin alone. The most potent effects were observed with the aqueous extract and the lowest with the pure saponin fraction. The tested triterpenoid saponin with an aldehyde group attached at triterpene position 4 enhanced the transport of RIPs through the cell membrane.	[35]
Cytotoxicity against cancer lines	RIP isolated from seeds (sodium salt of agrostin)	Agrostin inhibited the ^3^[H]-thymidine incorporation in HL-60 cells (human leukemic HL-60) in a dose- and time-dependent manner. The thymidine incorporation was reduced by 17.2% after a 72 h incubation with 10 μg/mL of agrostin. The incorporation of thymidine was reduced by 40.9% after a 120 h incubation with 30 μg/mL of agrostin. After 72 h of HL-60 cell incubation with 30 μg/mL of agrostin, the viable cell count was reduced by 43.9% and the number of necrotic and apoptotic cells increased by 100% and 244.8% of the control levels, respectively. At 10 and 30 μg/mL of RIPs, the proportion of sub G_1_ cells was increased by 290.4% and 756.7%, respectively. After 72 h with 30 μg/mL agrostin, the number of bcl-2-positive cells was reduced by 30.1%.	[34]
Protective effect against oxidative stress	Extracts from aerial parts	An extract from *A. githago* enhanced the antioxidant status and decreased the free-radical-induced lipid peroxidation in the blood of albino Wistar rats exposed to X-radiation. The cholesterol level in the group fed with a corncockle extract at a dose of 200 mg/kg was decreased from 2.57 ± 0.13 to 2.3 ± 0.19 mmol/L (after 21 days) and from 2.69 ± 0.16 to 2.3 ± 0.19 mmol/L (after 42 days). The triglyceride level in the group fed with the corncockle extract at a dose of 100 mg/kg was decreased from 2.0 ± 0.27 to 1.63 ± 0.14 mmol/L (after 21 days) and from 2.08 ± 0.54 to 1.60 ± 0.17 mmol/L (after 42 days). In the case of the group fed with the 200 mg/kg extract, the level of triglyceride decreased from 2.00 ± 0.27 to 1.67 ± 0.17 and 1.55 ± 0.18 mmol/L (after 21 and 42 days, respectively).	[37]
Anti-angiogenic effect	Aqueous seed extract	The extract down-regulated the expression levels of vascular endothelial growth factors 1 and 2, which resulted in decreased levels of metalloproteinase 2 and angioprotein 2. The saponin-rich extract of *A. githago* reduced human umbilical vein endothelial cell (HUVEC) and fibroblastic cell survival. The extract limited the growth of HUVECs in concentrations from 1 to 50 μg/mL. In 1–10 μg/mL, a cytotoxic effect was observed. In 20–50 μg/mL, a cytocidal effect was observed. The IC50 values for endothelial cells were 11.1 ± 0.5 μg/mL (48 h) and 10 ± 0.5 μg/mL (72 h).	[43]
Inhibition of *Leishmania major* culture growth	Aqueous seed extract	The inhibitory effect of the extract (IC_50_) was stronger than the positive control megulumine antimoniate (0.365 and 71.01 mg/mL, respectively).	[40]
Antihypercholesterolemic activity	Ethanolic and aqueous extracts of aerial plants	The level of malonyldialdehyde (in comparison to the control group) was lower in rats receiving 100 and 200 mg/kg *Agrostemma* extracts via gastric gavage for 20 days (8.89 ± 1.0 and 8.57 ± 0.5 μmol/L, respectively; control group: 9.48 ± 0.54 μmol/L). The opposite results were observed with glutathione (730.7 ± 80.2 and 743.8 ± 68.5 mg/L; control group: 687.3 ± 50.0 mg/L), vitamin C (22.3 ± 2 and 23.0 ± 1.1 mg/L, respectively; control group: 21.1 ± 5.1 mg/L), β-carotene (85.7 ± 10.6 and 92.6 ± 31.4 μg/L, respectively; control group: 61.5 ± 8.6 μg/L), and retinol (498.3 ± 56.0 and 547.6 ± 94.7 μg/L, respectively; control group: 430.5 ± 55.3 μg/L). The highest total antioxidant activity (AOA) was observed with the corncockle extract (200 mg/kg): 1.14 ± 0.1 mmol/L (control group: 0.86 ± 0.05 mmol/L). Irradiated rats were treated with plant extracts. This test exhibited significantly lower plasma glucose concentration than untreated controls. The group treated with the *A. githago* extract had a significant difference in the total antioxidant activity in comparison to the control (100 mg/kg group: 1.01 ± 0.09 mmol/L, 200 mg/kg group: 1.09 ± 0.03 mmol/L, control group: 0.79 ± 0.03 mmol/L). After another 21 days after the irradiation, the total antioxidant activity of the group treated with 100 mg/kg of extract was 0.94 ± 0.09 mmol/L and treated with 200 mg/kg was 1.02 ± 0.09. The AOA of the control group was 0.74 ± 0.05 mmol/L.	[36]
Antiviral activity	RIPs purified from aqueous seed extract	RIPs isolated from the extract reduced the number of local lesions caused by the tobacco mosaic virus on the leaves.	[14]
Inhibition of protein synthesis	RIPs purified from aqueous seed extract	The median infective dose (ID_50_) of the isolated proteins was >100 μg/mL (inhibition tested on HeLa cells). Purified RIPs inhibited protein synthesis (tested on poly (U) directed polyphenylalanine) in rabbit reticulocyte lysate. The above compounds showed no unspecific proteinase or RNAase activity.	[14]

## Data Availability

During this review study, no original data were generated.

## Abbreviations

A549	lung carcinoma cell line
AGS	gastric cancer cell line
API	Active Pharmaceutical Ingredient
AS 1	agrostemmasaponin 1
AS 2	agrostemmasaponin 2
BCL-2	B-cell lymphoma 2
BGC-803	human gastric cancer cell line
EB/AO	Acridine Orange/Ethidium Bromide
EVC-304	human umbilical vascular endothelial cells 304
CM	carboxymethyl cellulose
FRAME	Fund for the Replacement of Animals in Medical Experiments
G1	Gap 1
G2/M	Gap 2/Mithosis
GRAS	Generally Recognized as Safe
HDL-C	high-density lipoprotein C
HepG2	human liver cancer cell line
HFL-I	human fetal lung fibroblast cell line
HPLC	high-performance liquid chromatography
HUVECs	human umbilical vein endothelial cells
IC50	half-maximal inhibitory concentration
ID_50_	median infective dose
LD_50_	half-maximal lethal dose
LDL-C	low-density lipoprotein C
MALDI-TOF–MS	matrix-assisted laser desorption and ionization–time-of-flight mass spectrometry
MTT	(3-(4,5-Dimethylthiazol-2-yl)-2,5-Diphenyltetrazolium Bromide)
PBS	phosphate-buffered saline
pI	isoelectric point
RIPs	ribosome-inactivating proteins
SDS-PAGE	sodium dodecyl sulfate polyacrylamide gel electrophoresis
TCM	Traditional Chinese Medicine
TLC	thin-layer chromatography
